# Advancements in the Use of Bacteriophages to Combat the Kiwifruit Canker Phytopathogen *Pseudomonas syringae* pv. *actinidiae*

**DOI:** 10.3390/v14122704

**Published:** 2022-12-02

**Authors:** Jinyan Luo, Dejiang Dai, Luqiong Lv, Temoor Ahmed, Lei Chen, Yanli Wang, Qianli An, Guochang Sun, Bin Li

**Affiliations:** 1 Department of Plant Quarantine, Shanghai Extension and Service Center of Agriculture Technology, Shanghai 201103, China; 2 Station for the Plant Protection & Quarantine and Control of Agrochemicals Zhejiang Province, Hangzhou 310004, China; 3 State Key Laboratory of Rice Biology, Ministry of Agriculture Key Laboratory of Molecular Biology of Crop Pathogens and Insects, Institute of Biotechnology, Zhejiang University, Hangzhou 310058, China; 4 State Key Laboratory for Managing Biotic and Chemical Threats to the Quality and Safety of Agro-Products, Zhejiang Academy of Agricultural Sciences, Hangzhou 310021, China

**Keywords:** infection, diversity, genome, kiwifruit canker, phage-based control

## Abstract

Over the last several decades, kiwifruit production has been severely damaged by the bacterial plant pathogen *Pseudomonas syringae* pv. *actinidiae* (Psa), resulting in severe economic losses worldwide. Currently, copper bactericides and antibiotics are the main tools used to control this bacterial disease. However, their use is becoming increasingly ineffective due to the emergence of antibiotic resistance. In addition, environmental issues and the changes in the composition of soil bacterial communities are also concerning when using these substances. Although biocontrol methods have shown promising antibacterial effects on Psa infection under in vitro conditions, the efficiency of antagonistic bacteria and fungi when deployed under field conditions remains unclear. Therefore, it is crucial to develop a phage-based biocontrol strategy for this bacterial pathogen. Due to the specificity of the target bacteria and for the benefit of the environment, bacteriophages (phages) have been widely regarded as promising biological agents to control plant, animal, and human bacterial diseases. An increasing number of studies focus on the use of phages for the control of plant diseases, including the kiwifruit bacterial canker. In this review, we first introduce the characteristics of the Psa-induced kiwifruit canker, followed by a description of the diversity and virulence of Psa strains. The main focus of the review is the description of recent advances in the isolation of Psa phages and their characterization, including morphology, host range, lytic activity, genome characterization, and lysis mechanism, but we also describe the biocontrol strategies together with potential challenges introduced by abiotic factors, such as high temperature, extreme pH, and UV irradiation in kiwifruit orchards. The information presented in this review highlights the potential role of phages in controlling Psa infection to ensure plant protection.

## 1. Introduction

Kiwifruit has a good reputation and is generally known as “the king of fruits” due to its flavor and nutritional properties, such as its high vitamin C content [1]. Currently, the global production of kiwifruit is about 1.5–1.6 million tons/year. China, Chile, Italy, Iran, and New Zealand are the main kiwifruit-producing countries, representing more than 90% of the world’s total kiwifruit production [2]. However, the bacterial canker has been considered the most devastating disease in kiwifruit production, caused by *Pseudomonas syringae* pv. *actinidiae* (Psa). Severe losses caused by this disease have been reported in several kiwifruit-growing countries [3,4,5]. Psa infection can cause various symptoms and, in severe cases, plant death, which significantly reduces the yield and quality of kiwifruit [6,7,8,9].

Psa was first isolated in Japan in 1984 [10]. It was subsequently reported worldwide in countries such as Korea, China, Italy, Portugal, Spain, France, Turkey, South America, New Zealand, Slovenia, Greece, Georgia, Switzerland, Chile, and Australia [3,9,11,12]. The severe outbreak of this disease may be partially attributed to the clonal propagation of kiwifruit plants, which makes the pathogen spread quickly via seedlings [13]. Notably, other reasons for its widespread include its high phenotypic and genetic diversity among the Psa isolates as well as the emergence of new virulent strains [14,15,16,17]. Indeed, Psa isolates have been classified into six biovars, designated biovars 1–6, based on virulence and biochemical characteristics [18,19,20]. Among them, biovar 4 strains were transferred to the new pathovar *actinidifoliorum* due to its lower aggressiveness, which is substantially different from the other Psa biovars [21]. Furthermore, Psa strains belonging to biovar 3 have been found to be involved in the global pandemic of kiwifruit cankers [22,23].

Psa has caused and is still causing severe worldwide economic losses [3,4,5,6,7,8]. For example, the land value of orchards growing the popular kiwifruit variety Hort16A has depreciated from 300,000 to 46,000 USD per hectare, resulting in enormous damage to the New Zealand economy [8]. Indeed, New Zealand exported the highest dollar value worth (USD 2 billion, accounting for 50.9% of total kiwifruit exports) of kiwifruit in 2021 (https://www.worldstopexports.com/kiwifruit-exports-by-country/ (accessed on 1 November 2022)). Meanwhile, Psa infection could be effectively alleviated by the characterization of the pathogen and the development of a sensitive detection method [24,25,26,27,28]. Furthermore, copper bactericide and streptomycin have been widely applied to reduce the damage of this disease. However, their effectiveness has been restricted due to the emergence of antibiotic resistance. Additionally, antibiotic residues in leaves and fruits, environmental pollution, and changes in soil bacterial communities have limited their application [29,30,31]. Due to these concerns and restrictions to its effectiveness, the use of streptomycin to control plant diseases has been banned in some European countries, such as Italy and Portugal [2]. Some other methods, including physical, agricultural, and biological, have also been used to control Psa [13,32,33,34]. For example, several antagonistic bacteria and their metabolites have exhibited promising in vitro antibacterial activity against Psa; however, their efficacy in controlling Psa infection was less consistent under field conditions [35,36,37,38]. Interestingly, there was an upsurge in interest in using phage therapy to control plant bacterial diseases [39,40]. Several phage products, such as AgriPhage™ and EcoShield™, have already been developed and commercialized in the market [1]. Therefore, phages are regarded as eco-friendly alternatives for controlling Psa infection in kiwifruit plants due to their environmental safety, high specificity for host bacteria, non-toxicity to plants and beneficial microflora, and ability to kill antibiotic-resistant bacteria [41].

This review summarizes the recent advances in the use of phages to control bacterial canker disease in kiwifruit, together with the potential challenges of phage therapy and its prospects.

## 2. Characterization of Psa Phages

### 2.1. Isolation of Psa Phages

Psa phages have been successfully isolated from different niches throughout the world [42,43,44,45,46,47,48,49,50,51,52,53]. For example, Yin et al. [41] isolated and purified 36 Psa phages from the kiwifruit orchards in the major production area of China, while Ni et al. [46,47] isolated and characterized lytic phages PN05 and PN09 of Psa from river water in Hangzhou, China. Furthermore, most of these isolated phages are lytic; for example, Frampton et al. [49] found that 258 out of 275 isolated phages exhibited lytic activity against Psa in New Zealand, while Park et al. [53] isolated a lytic Psa phage PPPL-1.

In addition, these isolated phages have been found to be of various morphological types, for instance, 6.3%, 41.7%, and 52.1% of the isolated Psa phages had the siphovirus, podovirus, or myovirus morphology, respectively, as reported by Huang et al. [1,54]. Furthermore, Liu et al. [42] isolated a new lytic phage, PHB09, from kiwifruit orchard soil in Sichuan, China, which should be grouped into a new genus as it does not possess any known characteristics of the myovirus groups. Although these phages had varied host ranges, recent studies have shown that the phages possess great potential for controlling kiwifruit bacterial canker pathogens in the orchard [42,46,52].

### 2.2. Morphological Characterization

According to morphological observation using transmission electron microscopy (TEM), the phages of the class Caudoviricetes account for the majority (>97%) of *Pseudomonas* phages [41]. Following the tail shape characterization, the phages of the class Caudoviricetes typically consist of three distinct morphological types. The phages with podovirus type are characterized by short tails, the myovirus phages are characterized by double-layered contractile tails, while the siphovirus phages are characterized by long, flexible tails [55]. Additionally, morphological observation of the phages’ heads revealed them to be either icosahedral or oblate in all families. Table 1 presents information on Psa phages, including the tail shape, head size, and total length, which are highly consistent with the characterization of phages belonging to the class Caudoviricetes. For example, both PN09 and PHB09 were considered as myoviruses based on the morphological characteristics, which were obtained through TEM observation [42,49].

Furthermore, the geographical origin may affect the distribution of Psa phages in the three morphotypes. For instance, the results of Yu et al. [50] indicated that myovirus and podovirus phages account for most of the isolated phages in Korea. Yin et al. [41] reported based on TEM observations that of the 36 isolated phages, 1 was a podovirus, 1 a siphovirus, and the remaining 34 were myoviruses. Similarly, Frampton et al. [49] reported that of the 24 Psa phages collected from various environmental samples in diseased kiwifruit orchards, 1 was a podovirus, 1 a siphovirus, and the remaining 22 were myoviruses, respectively, suggesting that both in Korea and New Zealand, myoviruses were dominant phages. On the other hand, Di Lallo et al. [51] found that siphovirus and podovirus phages accounted for the majority in Italy.

### 2.3. Host Ranges

The host range of a phage is the taxonomic diversity of hosts it can successfully infect. Understanding the host range is very important for using Psa phages effectively. Many studies have indicated that most Psa phages have a narrow host range for infecting the Psa pathogen but cannot infect other bacteria in the micro-environment due to their high specificity against a certain genus or even species of bacteria [42,48,52]. Generally, the narrow host range of phages causes the infection of only the pathogenic bacteria. For example, Ni et al. [47] found that phage PN09 could lyse all of the 29 Psa strains but could not lyse the other distantly related bacteria. Similarly, the results of the host range tests indicated that phage PHB09 was able to lyse biovar 2 and 3 strains of Psa but could not infect the other tested *Pseudomonas* sp. strains [42]. On the other hand, Flores et al. [48] revealed a difference in the host range of the Chilean Psa phages. For instance, phage CHF33 exhibited greater lytic activity against different Psa isolates than phage CHF1; however, all of the tested Psa isolates could be lysed by combining the selected phages.

In general, the host range of phages depends on the type of phage and host bacteria. For example, Psa isolates from Japan, New Zealand, Italy and Korea exhibited various sensitivity to phages with various titers [42,47,48,52]. The result of Di Lallo et al. [51] showed that fPSA1 was able to lyse Psa isolates but unable to lyse other pseudomonads, suggesting that the host range of this Psa phage was very narrow. In contrast, some phages have been reported to be able to infect combinations of the different Psa isolates from various geographical locations. For instance, Frampton et al. [59] reported that phage φPsa17 was able to infect a large range of Psa strains, including those isolated from Japan, Italy, New Zealand, South Korea, and some less virulent strains from New Zealand, which are different from the virulent Psa strains. Furthermore, Di Lallo et al. [51] revealed that the host range of phage fPSA2 was broader compared to phage fPSA1. Frampton et al. [49] found that some Psa phages were not only able to effectively lyse Psa and other *P. syringae* pathovars but also could infect other species of *Pseudomonas*, such as *Pseudomonas corrugate* and *Pseudomonas viridiflava*.

Obviously, an individual Psa phage has minimal or no influence on non-pathogenic species, particularly beneficial microflora, due to its narrow host range, suggesting that the isolated Psa phages are promising alternatives for controlling bacterial cankers in kiwifruit. On the other hand, Psa strains have also been found to be infected by some other phages. For example, the host range test indicated that phage φ6, in addition to infecting the original host *P. syringae* pv. *syringae* (lytic efficiency was considered as 100%), also infected Psa strains Cra-Fru 12.54 (lytic efficiency of 101.3%) and Cra-Fru 14.10 (lytic efficiency of 96.8%) [56]. Therefore, the host range test indicated that some other phages might also be able to be applied to control bacterial cankers in kiwifruit.

### 2.4. The Lytic Activity

The lytic activity of phages is often characterized by a one-step growth curve with many features, such as the latent period, the rise period, and the burst size. The lytic activity was positively associated with the burst size but negatively associated with the latent period and the rise period [41]. The burst size is particularly important because the number of viruses produced represents the potential for other cells to be infected. Following the one-step experiment, the lytic Psa phages exhibited an S-shape growth curve characterized by both a high burst size and short latency. This indicates that all the phages replicated effectively in host bacterial cells [41,47]. As shown in Table 2, there was a great difference in the latent period, the rise period, and the burst size among the Psa phages. For example, the lysogenic phage fPSA1 exhibited a long latency and rise period with a high burst size, while the lytic phage fPSA2 had a short latent period and rise period with a low burst size. To successfully manage plant diseases using either phages alone or as a cocktail, lytic activity is a prerequisite for the application of phage therapy [2]. Thus, these data indicate that all phages that lyse Psa strains were promising candidates for the phage therapy of kiwifruit cankers. Compared to the above-mentioned lysogenic phages, most lytic phages appear to be very effective in killing bacteria.

## 3. Genome Analysis of Psa Phages

The genomes of phages consist of either single- or double-stranded (ds) DNA or RNA, which can be classified as either lytic or temperate according to their life cycle. All phages of the class Caudoviricetes have a genome with ds DNA. As shown in Table 3, the genome of Psa phages could be either linear or circular. The genome size of Psa phages ranged from 40,472 bp to 305,260 bp, with the G+C content ranging from 43.1% to 60.44%. Estimates of the genome size using pulsed field gel electrophoresis (PFGE) indicated that the genome size of Psa173 was about 110 kb, the genome size of ΦPsa17 was about 30 kb, and the genome sizes of the other 21 phages were about 95 kb [49]. Interestingly, preliminary sequence data indicated that the size of the ΦPsa21 genome was about 300 kb, which is greater than that of other Psa phages. The phages with genome sizes larger than 200 kb were designated as Jumbo phages, evolutionarily divergent from phages with smaller genomes. Indeed, Jumbo phages have larger capsids and more genes than smaller phages. This genome size enables them to be less dependent on the replication mechanisms of their hosts. Interestingly, Wojtus et al. [58] recently found that the transcription of a Jumbo phage happens independently of the host bacteria by encoding their own RNA polymerases.

Psa phage φPsa17 has been identified as a member of the T7-like virus genus (now named as Podovirus) based on a combination of genomic and proteomic assays as well as cryo-EM morphological observation [49,59]. Interestingly, a genomic analysis suggested that all Psa phages from Chile are closely related and similar to T7-like phages, having high similarity with other Psa phages from different countries, such as phiPSA2 (φPSA2) from Italy, phage PPPL-1 from Korea, and phiPSA17 from New Zealand. [48]. Therefore, it can be inferred that there is a global distribution of Psa phages, which is consistent with the pandemic of Psa biovar 3 at a global scale. Furthermore, the PFGE data correlated well with the genomic assembly, indicating that PFGE could be regarded as a useful tool to help and confirm the assembly of phage genomes [49].

Following the genome-sequencing results, the Psa phages from Northern Italy could be divided into four groups based on the similarity of their sequences. In accordance with the phylogenetic analysis, psageA1 and psageB1 are considered two newly defined species of phages infecting Psa in the class Caudoviricetes [52]. Furthermore, phage PHB09 has been regarded as a novel genus in the class Caudoviricete based on phylogenetic analysis of the complete genome sequence and amino acid sequences of the conserved proteins [42]. To investigate more subtle differences in the DNA sequences of these phages, restriction fragment length polymorphism (RFLP) analysis was also carried out using restriction enzyme digestion. Based on the sizes of these bands, the Psa phages exhibited different size patterns. Indeed, the genomic DNAs of most phages were digested with restriction enzymes, such as *Nhe*I, Eco*RI*, and *Sph*I, while some phages could be digested with *Hha*I and *Msp*I. However, all of the tested phages could not be digested by the following enzymes: *Mnl*I, *Nco*I, *Eco*RV, *Sau*3AI, *Sph*I, *Rsa*I, *Stu*I, *Xho*I, *Dra*I, *Acc*65I, *Hin*fI, *Kpn*I, and *Tsp*45I. These results indicated high diversity among the Psa phages collected from kiwifruit orchards [48,50].

**Figure 1:**
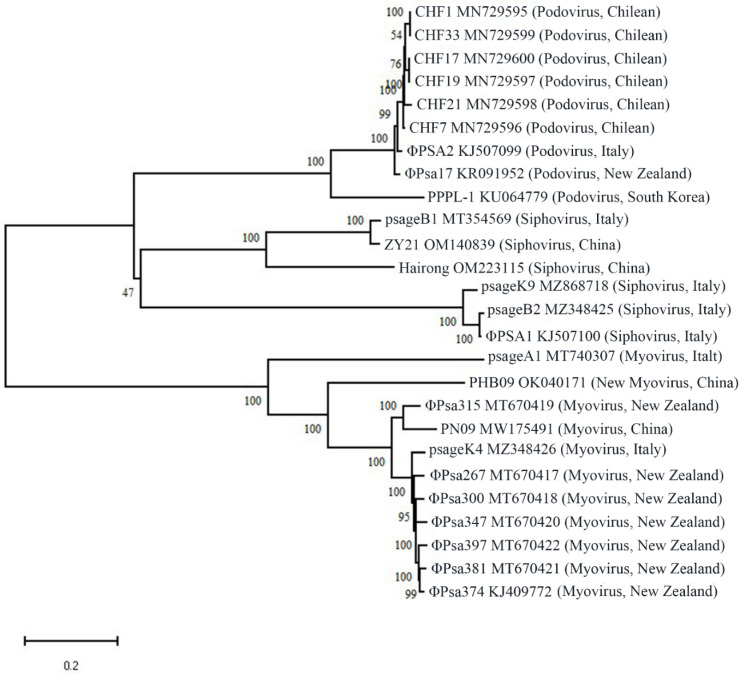
Phylogenetic tree of the Psa phages was constructed by MEGA 7.0 software using the maximum composite likelihood method based on the genome sequences available in the NCBI database. Nodes show the result of 1000 bootstrap replicates. The genome sequences were aligned using the MAFFT multiple sequence alignment (http://mafft.cbrc.jp/alignment/software/; accessed on 27 November 2022).

To investigate genetic diversity, we downloaded all Psa phage genomes available in the NCBI database. As shown in Table 4, these Psa phages originating from different countries differed in genome length, GC content, gene numbers, and classification. Interestingly, all Psa phages from Chile are podoviruses, while the Psa phages from China, New Zealand, and Italy are more variable. Furthermore, the genetic relationship of the Psa phages originating from different countries was revealed using the phylogenetic tree of the Psa phages, which was constructed based on the genome sequences available in the NCBI database using maximum composite likelihood (Figure 1). The result revealed that the Italian and Chinese strains exhibited greater diversity compared to the Chilean and New Zealand strains, which is generally consistent with the result of Table 4. However, in the same family, a very high similarity of genome sequences was observed among the Psa phages from different countries, which may be because they have the same origin. The wide distribution of phages in the same family may be mainly due to the international trade of kiwifruit seedlings. In addition, phylogenetic analysis of phages was also carried out in some studies based on large subunit terminases, which exhibited a similar result to that of genomes [60].

## 4. Infection Mechanism of Phages

The infection of host bacteria by a lytic phage involves a series of processes, which includes the attachment of phages to the host cells, injection of the DNA into the host cells, and self-replication in the host cells, leading to the death of host bacterial cells. The lytic activity of phages toward host bacteria has been, at least partially, attributed to endolysin and holin, which have been regarded as two lytic enzymes of phages [43,44,60]. Indeed, holins and endolysins have been widely reported to be able to damage the inner cell membrane and the peptidoglycan layer, respectively. For instance, it has been reported that phage endolysin LysPN09, produced by phage PN09, can cause the lysis of bacterial cells by effectively degrading the murein sacculus, which is the primary structural component of the cell wall in bacteria [47]. On the other hand, the function of endolysin is dependent on holin, which helps to transport the muramidases of the phage to the murein sacculus by perforating the inner cell membrane and determining the exact time point for the lysis of bacterial cells.

Recently, increased attention has been paid to finding new phage endolysins and their potential in agriculture as novel antibacterial agents [43,44,60]. Many studies have reported that Gram-positive bacteria are more sensitive to phage endolysins than Gram-negative bacteria. This may be because the peptidoglycan layers of Gram-negative bacteria cannot be degraded by endolysin due to the outer membrane layer. However, the lysis of phage endolysin on Gram-negative bacteria could be facilitated by EDTA, which has usually been used as an outer-membrane permeabilizer. For example, when combined with EDTA, the endolysin LysPN09 of phage PN09 was able to effectively infect all of the 29 tested Psa strains and exhibited strong activity on the Psa cells so that the outer membrane was permeabilized with good thermal and pH stability. On the other hand, some other mechanisms have been proposed for phage lysis [47]. For example, Ni et al. [46] recently found that the biofilm formation of Psa strains was effectively inhibited by the suspensions of either a single phage or a phage cocktail. The biofilm-removal mechanism is mainly attributed to the ability of phages to produce specific enzymes, which drives them to actively disturb and reduce the formation of the host’s bacterial biofilm. Furthermore, lytic phages can stimulate their host bacteria to produce more EPS-degrading enzymes, facilitating the penetration and movement of phages through the host’s bacterial biofilm. Subsequently, phages first penetrate into host bacterial cells via the biofilm, then proliferate within their host’s bacterial cells, and finally eliminate host bacteria via lytic activity.

## 5. Tolerance to Environmental Stresses

To effectively control the bacterial canker disease, it is necessary to check the activity of phages under various natural environments in kiwifruit-growing orchards, which is a key factor for effectively controlling phage therapy. Generally, Psa phages are negatively affected by various environmental conditions such as high temperatures, extreme pH, and ultraviolet (UV) radiation [45,47,50,51]. Although phages could still serve as a biocontrol agent of bacterial cankers, their population reduction by these factors has limited their efficiency and application in the field [41,61,62,63,64]. The hypothesized mechanisms of these abiotic factors have included that Psa phages are inactivated by damage to their structural elements and/or the promotion of DNA structural changes, which results in a decline in phage titers in the phyllosphere [2]. Notably, the reduction in phage activity depends on the specific strain of the phage, in which some were more tolerant than others [48]. Therefore, the utmost attention should be paid to the environmental adaptability factors of phages during their selection for the biocontrol of kiwifruit bacterial canker disease in the field.

Among these environmental factors, temperature and pH have been reported to play an important role in the survival and stability of phages by influencing the attachment, penetration, intracellular replication, and amplification of particles within host bacterial cells [2]. When the temperature is low, only some phages can inject their genetic materials into host bacterial cells, while when the temperature is high, the capsid proteins can be degraded, resulting in a longer phage latency period [42,56]. Furthermore, the extreme pH values prevented the attachment of phages to receptor sites of host bacterial cells by interfering with either the lysozyme enzyme or with other capsid proteins of phages [42]. In general, it has been reported that the optimum pH value for the lytic activity of most Psa phages and their proteins is between 6 and 8 [2,41,42,46,47,48,49,50,53,56]. However, as shown in Table 5, several Psa phages have been documented to tolerate a wide range of pH values, ranging from 2.0 to 12.0.

UV irradiation has been widely considered the most crucial factor for the reduction and loss of phage activity in the natural environment by affecting the longevity of phages in the plant phyllosphere [44,61,62,63,64]. Indeed, UV radiation can directly damage free viruses by degrading the proteins of free phage particles, changing the nucleic acid structure, and reducing phage infectivity [2]. In particular, the irreversible effect induced by shorter wavelengths was found on the genomic material, which resulted in both the modification of viral proteins and the formation of lethal photoproducts [2]. Compared to RNA phages, DNA phages are generally more sensitive to UV radiation due to the formation of lethal photoproducts such as thymine dimers induced by UV radiation, while dsDNA or dsRNA phages exhibited greater resistance to UV radiation than ssDNA or ssRNA phages [2]. However, the sensitivity of phage particles to UV radiation can be overcome by using different measures, such as high-titer phages in the morning or at night when radiation is limited [42].

## 6. Application in Disease Control

### 6.1. Lytic Activity

Several Psa phages that exhibited strong lytic activity against host bacteria have been successfully isolated in many different countries, such as Japan, Italy, New Zealand, Korea, and China [41,53]. In particular, these obtained Psa phages exhibited many beneficial characteristics, such as great lytic activity against host bacteria, little or no influence on other soil bacteria, and resistance to various environmental stresses. For example, Yin et al. [41] indicated that the 36 obtained phages have high specificity for Psa strains belonging to biovar 3. Furthermore, Yu et al. [50] revealed the stability of five Psa phages to different pHs and exposure to UV-B. Ni et al. [47] reported that phage PN09 was not only able to lyse all 29 Psa strains belonging to biovar 3 but was also stable under various temperature and pH conditions. These results indicated that most of the reported isolated Psa phages exhibited great lytic activity against Psa and environmental stability under in vitro conditions, suggesting that Psa phages from different geographical origins could serve as an alternative to control Psa infections in kiwifruit plants.

### 6.2. Biocontrol Potential

Several studies have highlighted the potential of Psa phages for the biological control of Psa infection in kiwifruit plants and orchards [50,52,57,65,66,67,68]. For instance, Flores et al. [48] isolated four Podovirus phages, CHF1, 7, 19, and 21, and found that these phages used alone or in combination caused a significant decrease in Psa symptoms under greenhouse conditions. The results of [42] indicated that phage PHB09 possesses good environmental stability, and it efficiently controlled bacterial cankers in kiwifruit, indicating that it has great potential for use in the biological control of Psa infection. Furthermore, Pinheiro et al. [56] found that the phage phi6 reduced the cell concentration of different Psa strains in lab conditions. Flores et al. [48] showed that the four phages isolated had great potential for the biological control of Psa infection under greenhouse conditions. Park et al. [53] revealed that the Podovirus phage PPPL-1 was effective against 16 of the 18 tested Psa strains and most of the tested pathovars of *P. syringae*. Song et al. [69] reported that the Podovirus phage PPPL-1 isolated from Korea was able to effectively control bacterial cankers in kiwifruit. In addition, phage PPPL-1 exhibited a similar inhibitory effect on bacterial cankers in kiwifruit plants compared with a commercial antibiotic-based product under greenhouse conditions [69]. Thus, it can be inferred that the lytic DNA phage might be a promising alternative for controlling bacterial cankers in kiwifruit plants.

### 6.3. Phage Cocktail

A host range analysis exhibited that Psa strains from different geographical origins could be lysed by most isolated Psa phages [42]. For example, Flores et al. [48] showed that six selected podovirus phages were very efficient at lysing different Chilean Psa isolates. Meanwhile, Pinheiro et al. [57] indicated that the pretreatment of phages KHUΦ34, KHUΦ38, and PPPL-1 was able to lyse biovar 2 and 3 cells of Psa strains and other pathovars *of P. syringae*. Furthermore, Frampton et al. [59] indicated that the host range of phage φPsa17 was relatively broader, capable of lysing Psa strains from New Zealand, Japan, Italy, and South Korea. Furthermore, the other pathvars of *P. syringae* and other *Pseudomonas* phages were also able to lyse cells of some Psa strains, which suggests that these phages could be used as suitable candidates for future phage cocktails against Psa infection. For example, the results of Pinheiro et al. [56,57] showed that the commercially available phage Φ6, besides infecting its original host Pss, also lyses strains Cra-Fru 12.54 and 14.10, which belong to biovar 3 of Psa. However, in some cases, the isolated phages were highly specific to certain Psa strains belonging to biovar 3, which greatly restricted the application of the phages in the control of kiwifruit bacterial canker.

On the other hand, the emergence of phage-resistant bacteria is a major challenge in the application of phages for the control of Psa infection. Several studies have shown that the use of either phage cocktails or combined therapies can not only increase the effectiveness of phage therapy but also prevent the emergence of bacterial resistance to phages [70]. For example, Song et al. [69] reported that bacterial canker disease in kiwifruit could be effectively suppressed when applying phage PPPL-1 in combination with KHUΦ34 and KHUΦ38, while the pretreatment of PPL-1 phage could efficiently control bacterial cankers in kiwifruit as much as the treatment of the antibiotics product. Flores et al. [48] observed that four phages (CHF1, 7, 19, and 21) isolated from Chile, individually or combined in a cocktail, have great potential for the biological control of Psa infection in kiwifruit plants, while the cocktail of phages was able to reduce the Psa load in kiwifruit leaves by more than 75% in comparison with untreated plants after 24 h of infection with Psa. Furthermore. Ni et al. [46] revealed that combining a phage cocktail (PN05 and PN09) with carvacrol exhibited higher efficacy when lysing Psa cells in vitro and prevented the emergence of resistant host bacteria. Obviously, compared to the phage cocktail alone, carvacrol significantly increased the efficacy of the phage cocktail in inhibiting bacterial growth [46]. These findings suggest that a phage cocktail is a promising alternative for the control of Psa infection in kiwifruit. A hypothesized mechanism is that the phages used to infect the host lead to phage synergy in killing the host [71].

### 6.4. Advantages

Due to the narrow host range, phages are considered very promising, safe, and efficient alternatives for preventing bacterial cankers in kiwifruit plants [2], unlike copper-based bactericides and antibiotics. Phages are one of the most abundant types of biological entities on Earth, with an approximate population of 10^30–31^ present in the biosphere. Due to their natural, ubiquitous presence in the biosphere [2,42], it is very easy for us to isolate phages that could potentially be used to control plant pathogenic bacteria [72]. Notably, most phages of Psa were unable to lyse other *Pseudomonas* species and other bacterial species collected from the natural environment of kiwifruit plants. However, several other authors have reported that Psa phages could lyse other *P. syringae* strains, even other species of Pseudomonas. As specific pathogen killers, phages have been considered to be relatively safe due to having no harmful effect on plant cells and other beneficial microorganisms [73]. On the other hand, a broader host range, which could serve as a potential synergy between phages, could be achieved by designing phage cocktails against other plant pathogenic bacteria in the species complex of *P. syringae* [47].

## 7. Challenges

Phage therapy has been regarded as an effective measure to control Psa infections in kiwifruit plants. However, applying Psa phages in plant protection still poses several challenges and limitations.

### 7.1. Resistance

The development of bacterial resistance is a main limiting factor in the application of phages to control Psa infection. For example, Pereira et al. [2] reported that Psa phages KHUΦ74 and KHUΦ59 were ineffective due to the emergence of bacterial resistance. The efficiency of phage as a biocontrol agent in orchards could be greatly reduced by the rapid evolution of phage resistance, which has been mainly attributed to the alteration/loss of the phage-binding site in the host’s bacterial lipopolysaccharide layer, flagella, pilus, or capsid [74]. Recent studies also found that phages could attenuate the virulence of bacterial pathogens, often driven by either the evolution of bacterial resistance against lytic phages or the infection of lysogenic phages [75]. Resistance can be acquired at all phage-infection stages (attachment to the host surface, penetration into host cells, transcription, biosynthesis, maturation, and lysis in the host cells) by either the selection of natural stresses or mutation caused by horizontal gene transfer [76,77]. The mechanisms of bacterial resistance to lytic phages include the adsorption prevention of the phage, blocking of DNA entry, restriction modification, abortive infection, and immune interference of CRISPR/Cas and modification-restriction systems [49]. In natural ecology, the frequency of bacterial resistance to phages is usually between 10^−6^ and 10^−8^, which is about 10 times lower than the frequency of bacterial resistance to antibiotics [2,48]. The emergence of bacterial resistance to phages can be overcome by applying phage cocktails containing genetically or morphologically different phages against Psa from various origins. The appearance of phage resistance in Psa has also been effectively prevented by applying carvacrol or endolysins to phage cocktails. Additional strategies to combat this pathogen include the combination of agricultural, biological, physical and chemical approaches, as well as the use of mutant, novel, or modified phages that are effective against the resistant Psa isolates.

### 7.2. Specificity

Previous studies have shown that the host range of Psa phages was very narrow, with no toxic effect on non-pathogenic bacteria in the micro-environment [2,25]. The high specificity makes a Psa phage only infect its target host bacteria, while different biovars of the Psa bacteria show alteration in phage susceptibility. However, in some cases, a high intraspecies diversity has been found in the targeted Psa pathogens due to local adaptation [54]. Interestingly, the application of phage cocktails will greatly expand the lytic range of host bacteria by selecting specific phages with a narrow host range. Furthermore, to develop customized phage cocktails that can infect the important plant pathogenic bacteria in agriculture, it is essential to set up a large collection of lytic phages [76]. In addition, novel phages should be frequently isolated and collected to obtain the phage resources that are very effective against newly emerging mutant and resistant strains. Current evidence suggests that a greater biocontrol effect could be achieved in kiwifruit bacterial cankers by mixing phages with other chemicals, such as streptomycin, or biological control agents together with SAR inducers, such as acibenzolar-S-methyl and harpin [74].

### 7.3. Formulation

The isolated Psa phages exhibited strong in vitro lytic activity under various stress conditions. However, as reported previously in the literature, most of the phages of plant pathogenic bacteria exhibited high sensitivity to abiotic factors, particularly UV radiation in the plant phyllosphere, microbial habits, and rhizosphere environments [16]. Indeed, in kiwifruit orchards, the plant surface is exposed to a high amount of UV radiation. Phages could be used in various formats, such as powder, solutions, and sprays. The use of phage formulations with protective compounds can extend the survival of phages under field conditions, especially UV radiation [78]. Furthermore, the successful application of phages will also depend on the time they are applied in the orchards. For example, it is better to use phages in the kiwifruit orchards at dawn or night, which will limit the harmful damage of the temperature, pH, and UV radiation and increase their persistence in the phyllosphere [78]. It was also reported that phages should be applied in early spring, before disease infection. In addition, it was reported that after 1 h of infection with Psa, a phage cocktail applied in kiwifruit plants significantly inhibited both the growth of host bacteria and the development of canker disease 30 days post-infection [46]. Due to the wide application of phage cocktails, the formulation of phage cocktails should be optimized to meet the requirement of the inoculums by guaranteeing high stability and purity. Indeed, the persistence of phage cocktails in the natural environment could be greatly increased by specific formulations, while their lytic activities could be improved by synergy with other compounds, such as carvacrol and garlic extract. Interestingly, the sensitivity of phages to various abiotic factors can be overcome by encapsulating them within nanocarriers or binding them to macroscopic supports to increase their stability and function—ideas that have attracted more and more attention in recent years [2].

### 7.4. Genetic Engineering

As more Psa phage genomes are sequenced [79], the biocontrol efficiency of phages is further increased using genetic engineering, which was recently proposed as an effective way to improve the activity of lytic phages. These new phages can be obtained using different techniques, such as chemical mutation, homologous recombination, phage recombineering of electroporated DNA, CRISPR-Cas gene editing, and in vivo recombineering using λ phages [80]. For example, the change in the host range of the commercially available phage φ6 has been mainly attributed to the mutation in its receptor-binding proteins [81]. The infection of phage φ6 to closely related *Pseudomonas* species may be due to the high mutation rate associated with its RNA-based genome, which allowed it to obtain some new infection niches [81]. In addition to infectivity, phages could also be engineered to better resist various environmental stresses, which increases their survival in orchards during their application. Although using genetically engineered phages is an option, it poses a risk to biological security since there is no control over the potential release of such genetically engineered phages into the environment.

## 8. Conclusions and Perspectives

Due to the extremely high abundance of phages in the natural ecology, phages have received increasing research interest as an alternative and eco-friendly approach for controlling Psa infection in kiwifruit orchards. The main limitations of the application of phage therapy in agriculture are their high sensitivity to UV radiation and the emergence of bacterial resistance to phages. However, this can be overcome by using phage formulation via micro- and/or nanocarriers, phage cocktails, or preadapted phages, as well as the adjustment of application timing to avoid UV radiation exposure. Interestingly, compared to phage-susceptible bacteria, phage-resistant bacteria grow slower and are less virulent to host plants. Additional strategies to increase the efficiency of phage therapy and prevent the development of bacterial resistance include the application of phages in combination with currently available biological, physical, and chemical treatments. Other strategies are using mutant phages obtained from the wild-type and isolating new or modified phages that exhibit strong lytic activity against the resistant bacteria. Furthermore, the survival of phages in the orchards could be increased using protective formulations and effective inoculation methods. In addition, the government and organizations should develop relevant legislation to guarantee the phages’ large-scale production and safe use. Although several studies have revealed that phages can be successfully used to control bacterial cankers in kiwifruit plants, more in vivo field experiments should be carried out to elucidate the ecological and evolutionary effect of phage therapy in the rhizosphere and phyllosphere. Additionally, a good knowledge of microbial interaction between the phage and host bacteria is necessary to produce an effective phage cocktail, which will be very helpful for applying Psa phages to kiwifruit orchards. In conclusion, phages have great potential to control bacterial cankers either alone or together with other control methods.

## Figures and Tables

**Table 1:** Morphological characterization of some Psa phages.

Phage Name	Region	Classification	Head Size (nm)	Total Length (nm)	References
φxwy0013	Shanghai, China	Siphovirus	73	252	[41]
φxwy0014	Shanghai, China	Myovirus	70	193	[41]
φxwy0026	Shanghai, China	Podovirus	80	102	[41]
CHF1,4,7,9–10,15–19,21,30,33	Chilean	Podovirus	60	/	[48]
ΦPsa1,21,267,268,281,292,300,315–317,331,343,347,374,375,381,386,393,394,397,410,440	New Zealand	Myovirus	67.1–126.1	167.7–293.1	[49]
ΦPsa17	New Zealand	T7-like Podovirus	55.9	55.9	[49]
ΦPsa173	New Zealand	Siphovirus	77.5	252.8	[49]
KHUΦ34	Korea	Myovirus	90	231	[50]
KHUΦ38	Korea	Podovirus	70	90	[50]
KHUΦ44	Korea	Myovirus	90	220	[50]
KHUΦ59	Korea	Podovirus	69	87	[50]
KHUΦ74	Korea	Podovirus	66	88	[50]
PHB09	Sichuan, China	New Myovirus	55.2	145	[42]
phiPSA1	Italy	Siphovirus	60	200	[51]
phiPSA2 (φPSA2)	Italy	Podovirus	60	/	[51]
PsageK4,K4e,A1,A2	Northern Italy	Myovirus	72	125	[52]
PsageK9,B1,B2	Northern Italy	Siphovirus	78	176	[52]
PN05	Zhejiang, China	Myovirus	/	/	[46]
PN09	Zhejiang, China	Myovirus	77.5	187.8	[46]
Φ6	DSMZ, Germany	Cystovirus	/	/	[56,57]
PPPL-1	South Korea	Podovirus	/	/	[53]
φPsa21	Jambo phage	Myovirus	/	/	[58]

“/”: data unavailable.

**Table 2:** The lytic activity of the representative Psa phages.

Phages	Lytic/Lysogenic	Latent Period	Rise Period	Burst Size (PFU/Host Cell)	References
φXWY0013	lytic	20 min	35 min	100	[41]
φXWY0014	lytic	15 min	35 min	200	[41]
φXWY0026	lytic	30 min	50 min	170	[41]
PN09	lytic	20 min	100 min	51.3	[47]
fPSA1	lysogenic	100 min	50 min	178	[51]
fPSA2	lytic	15 min	15 min	92	[51]
Φ6	lytic	100 min	20 min	60	[56,57]
PHB09	lytic	60 min	40 min	182	[42]

**Table 3:** Genomic characterization of the Psa phages from the currently available literatures.

Phage	Lytic/Lysogenic	Number of Genes	Size	G+C	References
PHB09	lytic	186 predicted genes, no tRNAs	94,844 bp	57.61%	[42]
PN09	lytic	177 predicted genes, nine tRNAs	99,229 bp	48.16%	[47]
fPSA1	lysogenic	52 predicted genes	51,090 bp	58.5%	[51]
fPSA2	lytic	47 predicted genes	40,472 bp	57.4%	[51]
psageA1	lytic	176 predicted genes, 14 tRNAs	98,780 bp	48.79%	[52]
psageB2	lysogenic	77 predicted genes, no tRNAs	50 kb	58.51%	[52]
PsageK4	lytic	179 predicted genes, 18 tRNAs	98,440 bp	60.44%	[52]
psageB1	lytic	161 predicted genes, 4 tRNAs	112,269 bp	56.47%	[52]
φPsa17	lytic	49 predicted genes, no tRNAs	40,525 bp	57%	[49,59]
φPsa374,	lytic	11 tRNAs	/	47.4%	[59]
Psa21	lytic, jumbo	420 predicted genes, 8 tRNAs	305,260 bp	43.1%	[49,58]
CHF1,7,19,21	lytic	48 predicted genes	40,557–40,999 bp	near 57%	[48]
phage φ6	lytic	/	/	/	[56]
φPsa173	lytic	/	~110 kb	/	[49]
φPsa1,267,268,281,292,300,315–317,331,343,347,375,381,386,393,394,397,410,440	lytic	/	~95 kb	/	[49]
φPsa374	lytic	173 predicted genes, 11 tRNAs	97,761 bp	47.4%	[49]

φPSA2 = fPSA2.

**Table 4:** Information of the Psa phages genomes available in the NCBI database.

Phage Name	Accession Number	Terminase Acc No.	Genome Length (Kb)	GC Content (%)	Gene Numbers	Morphotype	Countries
PN09	MW175491	QPB10483	99.299	48.16	177	Myovirus	China
CHF1	MN729595	/	40.999	57.3	49	Podovirus	Chilean
CHF7	MN729596	/	40.557	57.4	48	Podovirus	Chilean
CHF17	MN729600	/	40.882	57.3	48	Podovirus	Chilean
CHF19	MN729597	/	40.882	57.3	48	Podovirus	Chilean
CHF21	MN729598	/	40.557	57.4	48	Podovirus	Chilean
CHF33	MN729599	/	40.999	57.3	49	Podovirus	Chilean
ΦPsa267	MT670417	QNN99863	100.18	47.7	176	Myovirus	New Zealand
ΦPsa300	MT670418	QNO00040	99.27	47.7	171	Myovirus	New Zealand
ΦPsa315	MT670419	QNO00211	98.74	48.0	172	Myovirus	New Zealand
ΦPsa347	MT670420	QNO00383	99.69	47.7	174	Myovirus	New Zealand
ΦPsa374	KJ409772	AHJ87316	98.29	47.7	181	Myovirus	New Zealand
ΦPsa381	MT670421	QNO00557	98.8	47.8	173	Myovirus	New Zealand
ΦPsa397	MT670422	QNO00730	98.95	47.7	173	Myovirus	New Zealand
phiPsa17	KR091952	/	40.53	57.3	49	Podovirus	New Zealand
psageA1	MT740307	QNR53853	98.78	48.8	174	Myovirus	Italy
psageB1	MT354569	QOC57867	112.27	56.5	169	Siphovirus	Italy
psageK4	MZ348426	QXV71718	98.44	60.4	197	Myovirus	Italy
psageB2	MZ348425	QXV71641	50.74	58.5	77	Siphovirus	Italy
phiPSA1	KJ507100	AHZ95062	51.09	58.5	/	Siphovirus	Italy
phiPSA2	KJ507099	/	40.48	57.4	/	Podovirus	Italy
PHB09	OK040171	UAV84529	94.884	57.61	185	New Myovirus	China
PPPL-1	KU064779	/	41.15	57.0	49	Podovirus	South Korea
psageK9	MZ868718	UAW53939	51.47	58.5	87	Siphovirus	Northern Italy
ZY21	OM140839	UIS24573	112.01	56.5	169	Nickievirus	China
hairong	OM223115	UKL14915	112.842	55.1	173	Nickievirus	China

**Table 5:** Phage tolerance to various environmental stresses.

Temperature	pH	UV Irradiation and Solar Radiation	References
Most phages grow well at 4–25 °C	/	UV light affect phage stability	[49]
Five phages survive at 40 °C (1 h), reduce at 50 °C, inactivate at 60 °C	Most of the phages, survive at pH 3–11 (1 h), inactivated at pH 12	Most of the phages keep activity under 365 nm UV, reduced by >50% under 306 nm UV (60 min)	[50]
Phage PPPL-1 survive up to 40 °C	Can survive at pH 3–11	Can survive under UV-A	[53]
Phage φXWY0013, 0014 and 0026 survive at 25–60 °C, with optimum at 25–40 °C, inactivated at 70 °C	Can survive at pH 2–12	/	[41]
The majority of 13 phages survive at 37, 18, and 4 °C (1 h)	some phages were sensitive to pH 4 and 5, while other phages can endure these pHs	Most phages were sensitive to solar radiation (30 or 60 min) while some phages can endure.	[48]
PN09 was stable at 25–35 °C, a relatively strong activity at 45 °C, low activity at 55 °C, completely inactivated at 65 °C.	Survive well at pH 6.0–9.0, and could remain relatively high activity above a pH of 9.0.	/	[47]
Most phages can be stored successfully at 4 °C, and survival well at ambient temperature (25 °C).	/	/	[49]
Phage PHB09 can survive at 4–37 °C (12 h), decreased significantly at 37 °C and 50 °C (6 h)	Survive at pH 3–11 (1 h), significantly reduced at pH 3 and 11	Relatively high UV stability (0–60 min), with the increase of exposure time, the phage titers gradually decreased	[42]
Phage φ6 survive at 15 and 25 °C, completely inactivated at 37 °C (6 d)	Survive at 5 < pH < 10, the optimum pH is 6–8	The abundance of phage particles decreased when exposed to UV and solar radiation.	[46,56]
All phages showed a comparable titer at 4 °C, 26 °C and 37 °C (1 h), however, at 55 °C, some phages inactivated, while others remained stable and reduced activity	All phages could survive at pH 4–10 (18 h), but the lytic capacity was markedly decreased when expose to pH 2	The number of phages was reduced when exposure to UV-C irradiation (10 min), in most cases, the phage particles were completely inactivated (2 h)	[52]
fPSA1 and fPSA2 are viable when exposed to 40 °C (60 min), remain about 80% of viability at 50 °C (60 min); rapidly reduced at 60 °C and inactivated when exposure to 60 °C (40 min)	No reduction in lytic activity at pH 5–9 (1 h); the reduced activity at pH 10.0 and 11; and almost inactivated at pH 2 and 3	/	[51]

## Data Availability

All data supporting the conclusions of this article are included in this article.
